# Pain-Causing Venom Peptides: Insights into Sensory Neuron Pharmacology

**DOI:** 10.3390/toxins10010015

**Published:** 2017-12-27

**Authors:** Sina Jami, Andelain Erickson, Stuart M. Brierley, Irina Vetter

**Affiliations:** 1 Institute for Molecular Bioscience, the University of Queensland, St. Lucia, QLD 4072, Australia; sina.jami@uq.net.au; 2 Centre for Nutrition and Gastrointestinal Diseases, Discipline of Medicine, University of Adelaide, South Australian Health and Medical Research Institute (SAHMRI), North Terrace, Adelaide, South Australia 5000, Australia; Andelain.Erickson@sahmri.com (A.E.); stuart.brierley@flinders.edu.au (S.M.B.); 3 Visceral Pain Research Group, Human Physiology, Centre for Neuroscience, College of Medicine and Public Health, Flinders University, Bedford Park, South Australia 5042, Australia; 4 School of Pharmacy, The University of Queensland, Woolloongabba, QLD 4103, Australia

**Keywords:** animal venom, pain, ASIC, sodium channel, TRP channel, pore forming toxin

## Abstract

Venoms are produced by a wide variety of species including spiders, scorpions, reptiles, cnidarians, and fish for the purpose of harming or incapacitating predators or prey. While some venoms are of relatively simple composition, many contain hundreds to thousands of individual components with distinct pharmacological activity. Pain-inducing or “algesic” venom compounds have proven invaluable to our understanding of how physiological nociceptive neural networks operate. In this review, we present an overview of some of the diverse nociceptive pathways that can be modulated by specific venom components to evoke pain.

## 1. Venoms and Their Pharmacological Effects

Venoms can be defined broadly as toxins secreted by an animal for the purpose of harming or incapacitating predators or prey. Venoms are widely used by spiders, scorpions, reptiles, cnidarians, fish, and by a few mammals and marsupials. While some venoms are of relatively simple composition, many contain hundreds to thousands of individual components with distinct pharmacological activity [1,2,3,4]. Given the substantial metabolic production cost to venomous animals, it is therefore likely that these venom components confer some survival benefit, although it should be noted that this remains unclear for the vast majority of venom components.

Most commonly, venoms are delivered to the victim through wounds resulting from encounters with physical defence mechanisms such as claws, teeth, quills, spines and stingers. However, topical delivery of venoms, for example, to the eye has also evolved, in the case of spitting cobras, multiple times [5]. Broadly, venoms can be categorised as cytotoxic or neurotoxic; either mechanism can induce pain on envenomation, although many venoms are either non-noxious or even analgesic [5]. The latter include venoms that block voltage-gated calcium channels or voltage-gated sodium channels, either directly or indirectly via G-protein coupled receptors [1,6,7,8]. Paralytic toxins are often hypothesised to have evolved specifically for predatory purposes, while pain-inducing venoms are intuitively well suited for defensive purposes. Indeed, some of the most painful venoms—such as bee, wasp, stonefish or platypus venom—are only used for defensive or competitive purposes. However, paralytic effects and nociceptive activity are not mutually exclusive, suggesting that pain-inducing venom components may serve a dual purpose and that this simplistic classification is likely to be revised following advances in venom research. In addition, venomous animals are surprisingly adept at modulating the composition and amount of venom produced.

For example, cone snails such as *Conus geographus* are able to switch—rapidly, repeatedly and interchangeably—between venom containing high levels of paralytic toxins when faced with a human or predator, or venom containing prey-specific toxins that are mostly inactive at human targets when hunting for a meal [9]. Similarly, the spider *Cupiennius salei*, as well as several snakes, are able to adjust the amount of injected venom based on prey size [10,11]. Thus, the correlation of toxin abundance in dissected or extracted venom with pain reported after envenomation is likely confounded by erroneous assumptions about the evolutionary purpose of individual venom components with specific pharmacological activity.

Overall, algesic and analgesic venom compounds have proved invaluable to our understanding of how our physiological nociceptive neural networks operate. In this review, we present an overview of the nociceptive channels and neuronal pathways that are commonly modulated by specific venom components. Specifically, we highlight those venoms that induce nociceptive responses, and the diversity of mechanisms that these venoms target to evoke nociception.

## 2. The Neurobiology of Peripheral Pain Detection and Processing

Pain is indisputably a powerful motivator, and a crucial mechanism necessary for survival, as it allows an organism to alter its normal behaviours to protect the site of injury and facilitate proper tissue healing. Pain also encourages learnt avoidance behaviours that serve to prevent future exposure to noxious stimuli [12]. The evolutionary benefit of pain is convincingly illustrated by congenital insensitivity and indifference to pain, conditions arising from multiple genetic mutations that are associated with dysfunction of the nociceptive system. The resultant complete absence of pain sensations can lead to risk-seeking and self-mutilating behaviours that often lead to premature death as a result of trauma [13,14]. These incidents graphically illustrate the utility of pain for the defence of an organism against potentially life-threatening stimuli. Accordingly, activation of an organism’s nociceptive signalling system by venom peptides is a highly effective envenomation strategy.

**Figure 1:**
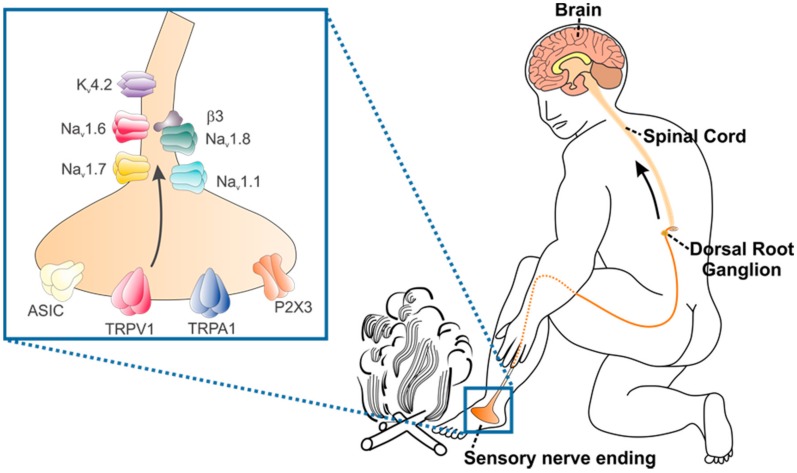
Pain due to envenomation typically occurs as a result of activation of nociceptive or “pain-sensing” peripheral nerve endings which express a host of ion channels and receptors that are targeted by venom components. Key molecular targets of algesic venom components include ASICs, TRPV1, TRPA1, Na_V_ and K_V_ channels. Activation of ASICs and TRPs contribute to generator potentials—small, graded depolarisation. K_V_ channels play key roles in setting the resting membrane potential, whilst once a threshold of membrane depolarisation has been reached, the opening of Na_V_ channels leads to the rapid upstroke of the action potential.

At the anatomical level, pain is typically the result of activation of nociceptive or “pain-sensing” peripheral nerve endings, which primarily consist of thinly myelinated Aδ and sparsely or unmyelinated C-fibre neurons [15]. The cell bodies of these neurons are located in the dorsal root ganglia, with the central branches of the axons terminating in the spinal cord, where they form synapses with second order neurons in the dorsal horn [15]. Signals originating from peripheral nociceptors further ascend via the spinothalamic as well as spinoreticular, spinomesencephalic and spinohypothalamic tracts for processing of pain perception in multiple higher brain centres [15]. Activation of nociceptors involves transduction of an external signal into a graded depolarisation of the nerve terminal. This typically occurs via a myriad of receptors and ion channels that respond to a range of chemical, mechanical, or thermal stimuli. For example, in recent years the Acid Sensing Ion Channels (ASICs) have emerged as key contributors to acid detection, whilst the Transient Receptor Potential (TRP) family of ion channels have been identified as key thermosensors encoding a broad range of temperatures from very cold to burning hot. Generator potentials—small, graded depolarisation—induced by activation of these ion channels in turn leads to the generation of action potentials, which transmit peripheral events along the axon of sensory neurons (Figure 1).

The threshold of action potential generation, or transformation of a nociceptive signal, is regulated tightly by ion channels involved in setting the resting membrane potential. Of particular importance in this regard are the potassium channels, in particular members of the voltage-gated (K_V_), two-pore domain (K2P) and inward rectifier (K_ir_) families (voltage-gated and K2Ps), as well as the Na^+^/K^+^-ATPase which maintains the required ionic gradients. To a lesser degree, hyperpolarisation-activated cyclic nucleotide-gated, voltage-gated Na^+^ channels (Na_V_), and voltage-gated Ca^2+^ channels (Ca_V_) also contribute to setting the resting membrane potential [16]. Once a threshold of membrane depolarisation has been reached, the opening of Na_V_ channels leads to the rapid upstroke of the action potential.

The biophysical processes of signal transduction, transformation and transmission offer a multitude of opportunities for venomous animals to elicit pain. In addition to the physical injury associated with a bite or sting (which may be minor), activation of Na^+^- or Ca^2+^-permeable channels or signalling pathways that lead to the downstream intracellular elevation of these ions results in membrane depolarisation and transformation of these generator potentials into action potentials. Alternatively, inhibition of ion channels that contribute to setting and/or maintaining the resting membrane potential can also achieve net excitatory effects, and thus induce pain. Often, venom components have dual activity and both activate Na^+^- or Ca^2+^-permeable channels and inhibit K^+^-permeable channels, causing synergistic activation of nociceptors, although it should be noted that for the vast majority of venom components, direct effects on sensory neurons and pain behaviours have not been assessed. This envenomation strategy effectively “hi-jacks” the body’s normal nociceptive pathways. In addition to specific modulation of molecular targets, a “molecular sledgehammer” approach to inducing pain is via pore-forming toxins, which allow indiscriminate ion flux. Specific toxins that act via some of these pathways are discussed in the following sections.

## 3. Voltage-Gated Sodium Channels

Voltage-gated sodium (Na_V_) channels are key transmembrane proteins that contribute to setting the resting membrane potential, and that are essential for propagation of action potentials in nociceptive neurons [17,18]. Nine subtypes, called Na_V_1.1–Na_V_1.9, have been identified and are encoded by the genes *SCN1A-SCN5A* and *SCN8A-SCN11A* [18]. Gain of function mutations that result either in enhanced activation or delayed inactivation have been associated with various conditions linked to enhanced pain, including paroxysmal extreme pain disorder and inherited erythromelalgia [7,19,20].

Although it is not a venom, the pan-Na_V_ channel activator ciguatoxin (P-CTX-1) is of interest as it causes ciguatera, the most common non-bacterial form of fish-borne illness in humans due to the consumption of fish contaminated with ciguatoxins [21,22] Key symptoms of ciguatera include heightened nociception, cold-allodynia and abdominal pain. Accordingly, ciguatoxin provides a key tool for comparison to venom based Na_V_ activators described below. Studies show that simultaneous activation of all Na_V_ channels by P-CTX-1 produces nocifensive responses when administered subcutaneously or intra-colonically in mice [21]. In mice, the somatosensory responses are likely mediated via Na_V_1.6 and Na_V_1.7 activation, as shown by inhibitory pharmacological modulation. In contrast, P-CTX-1 induced visceral pain appears to be predominantly mediated via Na_V_1.8 [21], highlighting the differing role of Na_V_ channels between somatic and visceral innervating nociceptors. In conjunction with these findings, researchers have discovered compounds in painful scorpion venoms that selectively activate Na_V_1.6 (Cn2) and Na_V_1.7 (OD1) [23,24,25,26]. Intraplantar injections of either purified venom peptide activates spontaneous pain behaviour, and, interestingly, activation of different pain modalities [23,24,25,26].

As Na_V_ channels are highly conserved across many phyla, the spastic paralysis induced by envenomation with Na_V_ activators has likely contributed to the evolutionary success of these compounds, resulting in convergent recruitment of this pharmacology.

Perhaps as a fortuitous coincidence—from the venomous animals’ perspectives—Na_V_ activators also typically elicit nocifensive responses after local injection. While subtype-selectivity for mammalian Na_V_ isoforms is likely not required as activation of at least Na_V_1.1, Na_V_1.6, Na_V_1.7 and Na_V_1.8 results in pain, structural similarities of mammalian Na_V_ isoforms to prey channels (e.g., fish and insect) in conjunction with differences between mammalian isoforms has led to the evolution of highly subtype-selective Na_V_ probes. Accordingly, Na_V_ channel activator toxins have been found in many venomous animals, including cone snails (δ-conotoxin SuVIA from *Conus suturatus*), spiders (e.g., δ-theraphotoxin-Hm1a from *Heteroscodra maculata*), sea anemone (e.g., ATX-II from *Anemonia sulcata*), scorpions (e.g., α-scorpion toxin CvIV4 from *Centruroides vittatus*), wasps (e.g., α-pompilidotoxin from *Anoplius samariensis*), and snakes (δ-elapitoxin-Cb1a from *Calliophis bivirgata*) (see Table 1) [27,28,29,30,31]. Generally speaking, such toxins achieve functional activation of Na_V_ channels through hyperpolarising shifts in the voltage-dependence of channel activation, depolarising shifts in channel inactivation, and through delaying the time-course of inactivation [21,32,33]. The net effect of these actions is typically enhanced neuronal excitability and activation of nociceptive pathways.

The recent discovery of δ-theraphotoxin-Hm1a (Hma1), a selective Na_V_1.1 modulator that impairs channel inactivation, revealed an unexpected role for Na_V_1.1 in mechanical pain [27]. Hm1a injection into the hind paw resulted in immediate nocifensive behaviours [27]. The genetic or pharmacologic elimination of TRPV1-expressing fibres greatly decreased sensitivity to heat, but did not affect sensitivity to mechanical stimuli, suggesting that Na_V_1.1 could potentially play a role in the transmission and transduction of mechanical stimuli [27]. Indeed, genetic deletion of Na_V_1.1 led to a decrease in nocifensive responses and decreased mechanical sensitisation [27].

Mechanical allodynia and hyperalgesia to colonic distension are crucial contributors to abdominal pain in patients with irritable bowel syndrome (IBS) [34,35]. Application of Hm1a enhanced mechanically-evoked firing in a sub-population of high-threshold colonic nociceptors in mice [27]. This effect was blocked by incubation with the Na_V_1.1/Na_V_1.3 antagonist ICA-121431 [27]. Furthermore, Hm1a also induces hyper-excitability of isolated colon-innervating DRG neurons from healthy control mice [27]. Importantly, colon-innervating DRG neurons isolated from a mouse model of IBS showed significantly enhanced responsiveness to Hm1a compared to healthy control mice, suggesting that Na_V_1.1 may be essential for the development and maintenance of chronic visceral pain conditions [27]. As such, antagonism of Na_V_1.1 may be a future target for the treatment of disorders accompanied by chronic visceral pain originating from the colon.

While systematic studies into the co-evolution of the pain system and pain-causing toxins are largely lacking, this evolutionary arms race is poignantly illustrated by the evolution and function of ion channels in specific species that frequent the habitat of venomous animals. For example, Grasshopper mice are resistant to the painful venom of bark scorpion which they prey on, due to a mutation in Na_V_1.8 [36]. Recently, pallid bats were also shown to be resistant to bark scorpion venom [37], providing key insight into binding sites, gating, and evolution of the channel.

Venom compounds that modulate Na_V_ channels have been extensively investigated for their potential to treat several forms of pain, and, although Na_V_ antagonists are outside the scope of this review, several excellent reviews of venom-derived Na_V_ antagonists are available (see [38]).

## 4. Transient Receptor Potential Channels

The transient receptor potential (TRP) channels belong to a superfamily of cation channels with diverse tissue distribution in multicellular organisms [39], and in humans includes 27 members in six subfamilies: TRPA(1), TRPV(1-6), TRPM(1-8), TRPP(1-3), TRPC(1,3-6), and TRPML(1-3) [40]. While TRP channels are expressed in almost every cell type in the body and have very diverse functions, they participate in nociceptive signalling primarily by relaying information on noxious temperature, chemicals, and pressure. Temperature detection is a particularly prominent role of TRPs, and it is generally thought that TRPV1-4 and TRPM2-5 channels respond to warm or hot temperatures, and that TRPA1, TRPM8, and TRPC5 respond to cool or cold temperatures, with preference for noxious or physiological temperature stimuli for each subtype within each category [40,41,42,43,44,45,46,47,48,49,50].

### 4.1. TRPV1

TRPV1 is activated by capsaicin, the active ingredient in hot chilli peppers, by noxious chemicals, and moderate heat under normal conditions. Interestingly, during in vitro pH conditions similar to that of algesic tissue acidosis, there is a shift in the activation threshold of TRPV1 to room temperature [39,51,52]. As a consequence of TRPV1 activation, there is a large influx of Ca^2+^, Na^+^, Mg^2+^ or Zn^2+^ ions, resulting in action potential generation in several classes of nociceptors. This mechanism is exploited by several venom-derived peptides acting at TRPV1, including RhTx from the Chinese red-headed centipede (*Scolopendra subspinipes mutilans*) [53], BmP01 from the scorpion *Mesobuthus martensii* [54], the selective and irreversible DkTx from the Earth Tiger tarantula *Ornithoctonus huwena* [55], venom components from the Palestine saw-scaled viper *Echis coloratus* [56], as well as vanillotoxins including VaTx3 from the tarantula *Psalmopoeus cambridgei* [57] (Table 2).

Interestingly, TRPV1 has relatively high structural similarity to the Shaker family of K_V_ channels, all of which are thought to form homo-or heteromultimeric proteins consisting of four six-transmembrane domain subunits. Several venom compounds that act as TRPV1 agonists have dual activity at voltage-gated potassium (K_V_) channels, perhaps suggesting that activity at TRPV1 is a natural extension of the pharmacology of venom peptides. Indeed, the vanillotoxins are closely related to K^+^ channel inhibitor toxins and both VaTx1 (τ/κ-theraphotoxin-Pc1a) and VaTx2 (τ-theraphotoxin-Pc1b) are known inhibitors of K_V_2.1 channels; this dual activity may contribute to pain-inducing effects in vivo [55].

While VaTx3 (τ-theraphotoxin-Pc1c) appears selective for TRPV1 over K_V_2.1, and no inhibition at K_V_1.2 was observed for any of the vanillotoxins, activity at other K^+^ channel isoforms cannot be ruled out at present [57]. Nonetheless, intraplantar administration of VaTx3 at >EC_90_ concentration (20 µM) caused TRPV1-mediated paw licking behaviours that were entirely abolished in TRPV1^−/−^ animals [57]. Interestingly, VaTx1 binds to distinct, non-homologous regions of the K_V_ and TRPV1 channels, suggesting that activity at TRPV1 is not simply a case of molecular and evolutionary pharmacology promiscuity [55]. Recently, it has been shown that the TRPV1-agonist scorpion venom peptides BmP01, Tx203, and OdK1 of the α-KTX8 scorpion peptide family all significantly potentiate TRPV1 under acidic conditions both in vitro and in vivo [58].

Venom peptides have also been crucial in the determination of the structure of TRPV1 and its distinct conformations in activate states [59,60,61]. Mutagenesis studies demonstrated that of the two putative extracellular protonation sites of TRPV1, BmP01 interacts with the outer pore and simultaneously occupies one of the protonation sites at E649. Mutation of the second protonation site E601 to alanine removed the enhanced activity of BmP01 during acidic conditions. More recently, DkTx was used to trap TRPV1 in an open configuration, enabling elucidation of the mechanisms of TRPV1 activation [62].

### 4.2. TRPA1

TRPA1 initiates pain signalling due to inflammatory mediators and noxious cold, and has a well-established role in toxin-induced pain [22]. Interestingly, no algesic venom peptide activator of TRPA1 has been described, although several peptides elicit analgesic effects via inhibition or activation-induced desensitisation of this channel [63,64,65,66].

The spider peptide ProTx-I (*Thrixopelma pruriens*) is analgesic in certain conditions, and a potent antagonist of several Na_v_ isoforms and TRPA1, possibly through interaction with the extracellular loops of the S1–S4 gating domain of both channel families [63]. Similarly, Phα1β (*Phoneutria nigriventer*) is a Ca_V_ and TRPA1 antagonist, and has been shown to attenuate mechanical and cold hyperalgesia induced by the TRPA1 agonist allyl isothiocyanate (AITC) in vivo [65]. On the other hand, the sea anemone venom peptide Ms 9a-1 is a positive modulator for TRPA1 in vitro and produces analgesic and anti-inflammatory responses in vivo, possibly via a desensitisation mechanism [66]. Similarly, crotalphine, a small venom peptide from a South American rattlesnake (*Crotalus durissus terrificus*), is a partial activator of TRPA1 and causes strong desensitisation of the channel, which may be responsible for its analgesic effect in vivo [64,67]. Taken together, these findings suggest that while TRPA1 is crucial for inflammatory and noxious cold signalling, it is not typically a key target of venom-induced pain. It is plausible that the paucity of venom peptide TRPA1 activators described to date arises from the unique signalling mechanism of TRPA1, which is robustly activated by reactive chemicals via modification of cysteine residues. Similarly, only indirect activation mechanisms have been reported for other TRP channel family members. For example, melittin-induced pain responses were reversed by the non-selective TRPC inhibitor SKF-96365, although neither direct channel activation nor off-target effects were assessed [68]. Given the important roles of TRP channels in sensory neuropharmacology, as well as the diversity of chemical and physical TRP channel activators, it is likely that further TRP modulators will be discovered from venoms in the future.

## 5. Voltage-Gated Potassium Channels

In the human genome, forty known genes encode the α subunits of voltage-gated potassium channels (K_V_) [69]. Similar to Na_V_ channels, K_V_ channels consist of four pore-forming α-subunits containing six transmembrane segments (S1–S6) that form a functional tetramer [70,71]. As some subfamilies of potassium channels (namely, K_V_1 or K_V_7) can form heteromultimers with other α-subunits, this results in considerable channel diversity with differing pharmacological and biophysical characteristics [72]. Sensory neurons express several K_V_ isoforms that repolarise the membrane potential following an action potential and are pathologically involved in hyperexcitability following injury and neuropathy [73,74,75].

The intrinsic function of K_V_ channels is to oppose membrane depolarisation or action potential generation and subsequent propagation [74]. Thus, K_V_ channels have an inhibitory role in sensory neuron excitability [74]. Decreased K_V_ activity is a hallmark of the hyper-excitability demonstrated in traumatic injuries and neuropathy [74].

Like Na_V_ toxins, venom peptides can block K_V_ function through occlusion of the pore, or by binding to the voltage-sensing domains to alter channel function [76]. K_V_ inhibitors have been isolated from the venom of many species, including cone snails (e.g., κM-conotoxin RIIIK from *Conus radiatus*), scorpions (e.g., Ts8 from *Tityus serrulatus*), spiders (e.g., κ-theraphotoxin-Gr1a from *Grammostola spatulata*), snakes (e.g., α-dendrotoxin from *Dendroaspis angusticeps*) and sea anemone (e.g., BDS-II from *Anemonia sulcata* [77,78,79,80].

Surprisingly, despite a clear role for K_V_ channels in regulating sensory neuron excitability (for review see [73]), the pain-inducing effects of K_V_ inhibitors have not been assessed systematically, albeit some K_V_ inhibitors have well-described effects on sensory neuron function. As an in-depth discussion of the role of potassium channels in pain pathways is beyond the scope of this review, the reader is referred to several excellent publications on the matter [73,75,81,82]. In brief, sensory neurons express many K_V_ isoforms, including K_V_ 1.1, 1.2, 1.3, 1.4, 1.6, 2.1, 2.2., 3.1, 3.2, 3.3, 3.4, 4.1, 4.3, 6.2, 6.4, 11.1, 10.2, 11.2, 11.3, 12.1, 7.1–7.5, 9.1, 9.3, and K_V_8.1 [83]. While the precise contribution(s) of these isoform to sensory signalling remain unclear, toxins with activity at these channels could be expected to lead to enhanced nociception. Indeed, dendrotoxin was shown to induce cold allodynia via K_V_1-mediated regulation of cold-sensitive trigeminal neurons in concert with TRPM8 [84]. Similarly, Ts8—a scorpion venom toxin that selectively inhibits K_V_4.2 over K_V_1.1–1.6, 2.1, 3.1, 7.1, 7.2, 7.4, 7.5, and K_V_10.1—elicited spontaneous nociceptive behaviour after intraplantar injection as well as mechanical allodynia after intrathecal injection [78]. In addition to providing an excellent defensive strategy, K_V_ channel inhibitor toxins will undoubtedly provide important research tools to unravel the complex pharmacology of these important ion channels.

## 6. Acid-Sensing Ion Channels

The Acid-sensing ion channel (ASIC) family contains six subunits (ASIC1a, ASIC1b, ASIC2a, ASIC2b, ASIC3 and ASIC4) encoded by four genes (ASIC1–4) [85,86]. ASIC1, -2, and -3 are highly expressed in the peripheral nervous system (PNS), where they are involved in detecting localised acidic pH changes and mediate acidosis-induced pain [86]. Whilst the roles of individual ASIC isoforms in nociception have been extensively studied using ASIC knockout mice, the function of homo- and heteromultimeric channel assemblies in pain pathways requires further investigation [85,86,87]. Recent evidence shows that at least three subunits are required to form a functional ASIC, where ASIC1a, ASIC1b, ASIC2a and ASIC3 can form homomultimers and heteromultimers with other ASIC subunits, the exception being that ASIC2b cannot form a homomultimer [87,88].

Many venoms are acidic, and it is thus not surprising that acid-sensitive channels such as ASICs might contribute to venom-induced pain either through proton-mediated activation or indirectly as a result of inflammation-induced tissue acidification. However, despite the prominence of ASIC channels in pain pathways, only one venom component that directly activates ASICs has been described so far—MitTx, a 20.8 kDa heteromeric toxin complex from the Texas coral snake *Micrurus tener tener* [89]. MitTx is composed of a Kunitz- and a phospholipase A2-like protein and elicits large, non-inactivating currents from ASIC1- and ASIC3-containing channels, as well as sensitising ASIC2a homomers to protons [89]. Indeed, nocifensive licking responses elicited by intraplantar injection of MitTx were virtually abolished in ASIC1^−/−^ mice, despite the injected concentration being orders of magnitude greater than the in vitro EC_50_ at ASIC1-containing channels (9.4–23.0 nM) [89]. While MitTx remains the only ASIC-activator toxin described to date, it is plausible that similar mechanisms are conserved across other species.

## 7. Phospholipase A2

Arachnid, insect, cnidarians, and snake venoms are particularly rich sources of phospholipase A2 (PLA2), an enzyme that catalyses the hydrolysis of phospholipids to produce arachidonic acid and lysophosphatidic acid [90,91]. Conversion of arachidonic acid to eicosanoids and leukotrienes facilitates activation of sensory neurons via inflammatory signalling cascades. The toxicity of secreted venom phospholipases A2 can vary significantly, involving complex pharmacological activities that ultimately lead to neurotoxic and/or myotoxic effects. Venom-derived PLA2s can be divided into structurally and functionally distinct subtypes: the snake-venom derived type 1 and 2 PLA2s; type 3 from *Heloderma suspectum*, *Heloderma horridum horridum* and *Apis mellifera*; and type 4, which consists of 40–80 amino acid residue peptides that are secreted by some cone snails.

Envenomation with snake venom PLA2s can have a range of biological effects, most notably neuromuscular toxicity, myotoxicity, cardiotoxicity, haemolytic and anti-coagulant effects, as well as organ and tissue damage [92]. Extracellular lysophospholipids and fatty acids, particularly lysophosphatidylcholine, recapitulate several aspects of the neuromuscular effects of envenomation with PLA2-containing venoms [93]. However, the role of venom-derived PLA2s in pain, as well as their effects on sensory neurons, is unclear. Mammalian PLA2 is an important contributor to inflammatory processes, with arachidonic acid derivatives produced downstream of enzyme activity contributing to sensitisation of sensory neurons, for example via activation of TRPV1 [94]. Accordingly, venom-derived PLA2s are also known to induce inflammation, and these processes most likely contribute to activation of nociceptors. However, enzymatically inactive venom-derived PLA2s are still able to elicit pain, suggesting that PLA2 as an inert molecule may activate nociceptive pathways [92,95,96]. Indeed, secreted PLA2s can (in addition to causing cytolytic effects and binding to M-type or other PLA2 receptors) directly block neuronal K_V_ channels, thus enhancing the duration of presynaptic membrane depolarisation [90,97,98]. This activity contributes to neuromuscular effects, and likely—as discussed above—also enhances excitability of sensory neurons [97]. This pharmacological promiscuity makes it difficult to unravel the precise contribution of PLA2s to pain resulting from envenomation.

For instance, melittin, a major pain-producing component from the venom of the honey bee *Apis mellifera*, is commonly described as a toxin mediating its effects via activation of PLA2. Indeed, melittin induces hyperactivity of PLA2, possibly as a result of Ca^2+^ influx [99]. Melittin, however, is a multifaceted toxin, as it also causes pore formation which may contribute to activation of nociceptors [100]. In addition, subcutaneous administration of melittin up-regulates voltage gated sodium channels Na_V_1.8 and Na_V_1.9, key ion channels involved in propagation of action potentials in sensory neurons [101].

## 8. Pore-Forming Venom Peptides

Pore-forming peptides are typically produced by bacteria, but are also found in many types of venoms, including cnidarians, ants, spiders, bees and wasps. These toxins are frequently cytolytic due to their ability to disrupt cellular membranes [102]. These peptides principally function based on their charge distribution as well as the phospholipid composition of cellular membranes [102]. Binding to target membranes can involve specific receptors such as cholesterol, sphingomyelin or membrane proteins, making some pore-forming toxins cell-type specific [103]. The proposed mechanism of pore formation involves a time-dependent build-up of the peptide in the lipid bilayer in a toroidal or barrel stave fashion which forms the pore [104]. In the toroidal mechanism, the pore-forming peptide accumulates and covers the surface of the outer phospholipid bilayer [102]. Subsequently, the peptides are integrated into the outer bilayer and expansion occurs in a manner that leaves the inner phospholipid bilayer smaller than the outer, with a toroidal pore forming by the bending of the inner bilayer [102]. Barrel stave peptides function in the same manner except they do not bend the inner bilayer [102]. The resultant transmembrane pores compromise membrane integrity and permit passage of ions and signalling molecules, leading to diverse biological effects.

Direct nociceptive effects have been shown for δ-myrtoxin-Mp1a, an α-helical peptide from the venom of the Jack Jumper ant *Myrmecia pilosula* [105]. Activity of Mp1a was initially assessed using dual-polarization interferometry, which showed disruption of phospholipid bilayers due to irreversible binding. This activity in turn results in a concentration-dependent transient increase in intracellular Ca^2+^ ions in vitro. Accordingly, local intraplantar injection of the toxin causes spontaneous pain as well as mechanical allodynia [105].

A similar mechanism also contributes to the pain-inducing effects of α-haemolysin, a pore forming toxin produced by *Staphylococcus aureus* [106]. α-Haemolysin is secreted by *Staphylococcus aureus* and is a known pore-forming toxin responsible for tissue damage, inflammation and bacterial migration. Insertion of α-haemolysin into cell membranes leads to formation of heptameric pores that allow cation influx, including in sensory neurons [106]. As a result, neuronal depolarisation leads to spontaneous action potential firing, an effect that is absent with a mutant α-haemolysin protein that does not form a stable pore [106]. In principle, any pore-forming toxin that is able to target the membranes of sensory neurons is likely to affect excitability of nociceptors and may thus lead to pain. In addition, cytolytic effects of these toxins could lead to lysis of non-neuronal cells in the skin and subsequent inflammatory activation of nociceptors. However, the contribution of these mechanisms to venom-induced pain has not been assessed systematically.

## 9. Conclusions

Studies focusing on the venoms produced by a myriad of species have provided crucial insight into prey–predator interactions. By isolating the thousands of different peptide components of these venoms, the field has provided new insights into venom-targeted channels and receptors. By taking advantage of millions of years of evolution, researchers have gained highly valuable insights into sensory function and pain signalling in physiological and pathological conditions. Finally, these venom-derived compounds have allowed elucidation of ion channel gating and activation mechanisms, and even species evolution of ion channels and their function.

## Figures and Tables

**Table 1:** Examples of pain-inducing venom peptides with activity at voltage-gated sodium channels (Na_V_).

Venom Peptide	Species	Pharmacological Target(s)	Pain Phenotype (Route)	Reference
δ-theraphotoxin-Hm1a	*Heteroscodra maculata*	Na_V_1.1	Spontaneous pain (i.pl.), mechanical allodynia (i.pl.)	[27]
OD1	*Odontobuthus doriae*	Na_V_1.7	Spontaneous pain (i.pl.)	[25]
Cn2	*Centruroides noxius*	Na_V_1.6	Spontaneous pain (i.pl.), mechanical allodynia (i.pl.)	[23]
δ-conotoxin SuVIA	*Conus suturatus*	Na_V_1.3, Na_V_1.4, Na_V_1.6, Na_V_1.7	Spontaneous pain (i.pl.)	[31]
α-scorpion toxin CvIV4	*Centruroides vittatus*	Na_V_1.2, Na_V_1.3, Na_V_1.4, Na_V_1.7	Spontaneous pain (i.pl.)	[24]

**Table 2:** Examples of venom peptide activators of TRPV1.

Venom Peptide	Species	Pharmacological Target(s)	Reference
RhTx	*Scolopendra subspinipes mutilans*	TRPV1	[53]
BmP01	*Mesobuthus martensii*	TRPV1	[54]
DkTx	*Ornithoctonus huwena*	TRPV1	[55]
*Echis coloratus* venom	*Echis coloratus*	TRPV1	[56]
VaTx1 (τ/κ-theraphotoxin-Pc1a)	*Psalmopoeus cambridgei*	TRPV1, K_V_2.1	[57]
VaTx2 (τ-theraphotoxin-Pc1b)	*Psalmopoeus cambridgei*	TRPV1, K_V_2.1	[57]
VaTx3 (τ-theraphotoxin-Pc1c)	*Psalmopoeus cambridgei*	TRPV1	[57]
